# Exploring the relationship between novel Coronavirus pneumonia and Parkinson’s disease

**DOI:** 10.1097/MD.0000000000031813

**Published:** 2022-11-18

**Authors:** Xiaoming Xi, Liang Han

**Affiliations:** a Rehabilitation Center,Beijing Rehabilitation Hospital Affiliated to Capital Medical University, Beijing; b Shandong University of Traditional Chinese Medicine.

**Keywords:** Coronavirus disease 2019, Parkinson’s disease, pathological mechanism, telerehabilitation

## Abstract

The hypothesis is that there is 0a relationship between Parkinson’s disease and coronavirus disease 2019 (COVID-19). By summarizing the pathogenesis of Parkinson’s disease and COVID-19 and the impact of COVID-19 on the central nervous system, the relationship between Parkinson’s disease and COVID-19 was analyzed, including whether Parkinson’s disease is a predisposition factor for COVID-19 and whether COVID-19 causes the occurrence of Parkinson’s disease. Discuss the impact of COVID-19 on patients with Parkinson’s disease, including symptoms and life impact. To summarize the principles, goals and methods of home rehabilitation for Parkinson’s disease patients during COVID-19. Through the analysis of this paper, it is believed that COVID-19 may cause Parkinson’s disease. Parkinson’s disease has the condition of susceptibility to COVID-19, but this conclusion is still controversial.

## 1. Introduction

Coronavirus disease 2019 is a respiratory infection characterized by dyspnea, fever, and cough, which started in 2019 and became a pandemic in March 2020.^[1]^ The new coronavirus, severe acute respiratory syndrome Coronavirus-2 (SARS-CoV-2) can spread beyond the respiratory system, causing intravascular coagulation and thrombosis, leading to acute renal failure and ultimately multi-organ failure.^[2]^ A significant number of coronavirus disease 2019 (COVID-19) patients may suffer from central nervous system disorders (headache, fatigue, stroke, dizziness, syncope, epilepsy)^[3]^ and peripheral nervous system disorders (absence, anosmia, neuropathic pain, and myalgia).^[4]^ Neurological symptoms were also observed. It may also affect the clinical course of several chronic neurological diseases, especially in elderly and frail patients, including Parkinson’s disease (PD).^[5]^ However, it has been suggested that dopamine may be involved in the pathophysiology of COVID-19,^[6]^ and amantadine and *α*-synuclein may have protective effects against COVID-19.^[7,8]^ For example, *α*-synuclein around the neuronal expression in the nervous system inhibits viral invasion of the central nervous system (CNS)^[9]^ and limits RNA virus replication in the brain,^[10]^ and physiological *α*-synuclein also recruits immune cells and protects against other infections,^[11]^ which play a role in pro-inflammatory responses. Therefore, whether and what kind of pathological mechanism link exists between COVID-19 and Parkinson’s remains controversial. It is unclear whether PD is a risk factor for SARS-CoV-2 infection and whether COVID-19 can cause PD. Therefore, this paper reviews the existing research and analyzes the relationship between the 2 to provide a reference for subsequent research.

### 1.1. The effects of COVID-19 on the central nervous system

COVID-19 is associated with polyneuropathy,^[12]^ encephalitis,^[13]^ and stroke,^[14]^ with common CNS symptoms, including hypofunction, headache, weakness, and altered consciousness et al^[15,16]^ Studies have shown that after the acute infection period of COVID-19, the new coronavirus RNA persists in the central nervous system, invades neurons or glial cells,^[17]^ and causes an immune response,^[18]^ resulting in neuronal loss.^[19]^ There is additional evidence that angiotensin-converting enzyme receptor 2 (ACE2) is expressed in glial cells and neurons in the brain after SARS-CoV-2 infection and is activated. It is increased in type 1 macrophages (M1) and tissue-specific macrophages,^[20,21]^ which can mediate viral entry, initiate intracellular inflammation, and induce ACE2 receptor shedding.^[22]^ One or more factors of viral encephalitis, systemic inflammation, peripheral organ dysfunction, and cerebrovascular alterations expose survivors of COVID-19 to chronic neurological diseases for a long time.^[23]^ Approximately 1 to 3rd of COVID-19 patients recover with cognitive and motor impairments, possibly related to systemic inflammation following COVID-19 infection, leading to cognitive decline and neurodegenerative changes,^[24,25]^ suggesting that survivors of COVID-19 may experience long-term neurodegenerative disease. Pulmonary dysfunction such as dyspnea occurs after SARS-CoV-2 infection, and chronic ventilation has become a common treatment for COVID-19. However, studies have shown that chronic ventilation is highly associated with persistent cognitive decline, executive dysfunction, and decreased quality of life,^[26]^ chronic ventilation may be associated with neurodegenerative changes, but the specific mechanism has not been elucidated. Patients with COVID-19 experience gastrointestinal symptoms such as anorexia, vomiting, nausea, abdominal pain, diarrhea, and bleeding.^[27,28]^ Gastrointestinal symptoms are closely related to the quantity and type of intestinal flora^[29,30]^; therefore, patients with COVID-19 may experience intestinal flora disturbances. The gut microbiota is closely related to the brain through the brain-gut axis,^[31–33]^ and changes in the gut microbiota are also considered an important cause of neurodegenerative diseases.^[34–38]^ It promotes the overexpression of *α*-synuclein in the gut and transports it to the brain to form deposits through the brain-gut axis,^[39]^ activates microglia to secrete pro-inflammatory factors, causes neuroinflammation, and induces or exacerbates neurodegenerative diseases.^[40]^ In addition, gut microbiota can also lead to lesions by regulating the activation threshold of the immune response and reactive T cells, promoting Th1 and Th17 cells to cross the blood-brain barrier and migrate to the brain.^[40]^ Therefore, COVID-19 may indirectly lead to central nervous system diseases by disrupting the homeostasis of the intestinal flora.

According to a review, SARS-CoV-2 infection can cause CNS damage through a variety of mechanisms, including direct infection of neurons, glial, or endothelial cells within the nervous system, resulting in acute cell death, and persistent central and peripheral damage in the cellular repertoire of the nervous system, leading to chronic inflammation, potentially progressive neurological damage, and autoimmune damage.

### 1.2. Pathogenesis of PD

PD is the second leading neurodegenerative disease after Alzheimer’s disease, and it is estimated that by 2040, there will be 12 million patients with PD worldwide.^[41]^ There is no unified conclusion on the etiology of PD, but it is mostly believed to be related to the degeneration of substantia nigra dopaminergic neurons and the formation of Lewy bodies.^[42]^ The pathogenesis of PD is complex and involves the nervous, digestive, immune, and other cellular and molecular pathological mechanisms. For more details, see Table 1.

**Table 1:** Pathogenesis of Parkinson’s disease.

Causes of disease	Pathogenesis	Reference
Abnormal aggregation of *α*-synuclein	Misfolded into amyloid form, involved in Lewy body composition; aggravates mitochondrial dysfunction, causes oxidative stress, activates neuroinflammation, causes or aggravates UPS abnormalities, induces axonal damage	^[43,44]^
Oxidative stress	Destabilizes nucleic acids; promotes *α*-synuclein, Parkin aggregation, and proteasome breakdown	^[45,46]^
Mitochondrial dysfunction	Disruption of mitochondrial dynamics, bioenergetic defects, complex I inhibition, ETC and increased ROS content; mitochondrial protein phosphatase and PTEN-induced PINK1 and Parkin function and induction of neurodegeneration	^[47,48]^
autophagy	Degradation of misfolded proteins and damaged organelles by lysosomes	^[49–51]^
Neuroinflammation	Promotes alpha-synuclein synthesis and aggregation; induces abnormal innate and adaptive immune responses	^[52]^
Microbial-intestinal-brain axis regulatory mechanisms	Induces alpha-synuclein misfolding and translocation of alpha-synuclein to the brain; inhibits glial cell activation; produces neuroinflammation	^[53,54]^
UPS dysfunction	Induced abnormal protein aggregation, striatal damage, mitochondrial autophagy, and increased DNA content	^[55–57]^
Excitatory neurotoxicity	Increased glutamate leads to oxidative stress and excitotoxicity, affecting cell viability and contributing to neuronal death	^[58,59]^
Genetic factors	Genetic mutations	^[60,61]^
Environmental risk factors	Associated with pesticide and heavy metal exposure, rural life, agricultural occupations, head trauma, melanoma, dairy consumption, type 2 diabetes, etc., with no conclusive mechanism	^[62–64]^

ETC = electron transport chain, ROS = reactive oxygen species, UPS = Ubiquitin - proteasome system.

### 1.3. Possible relationship between COVID-19 and PD

As discussed above, COVID-19 can directly or indirectly lead to central nervous system lesions. PD is a typical degenerative disease of the central nervous system. Therefore, it can be deduced that COVID-19 may induce PD. Experiments in mice suggest that SARS-CoV-2 may increase the risk of brain degeneration and symptoms of PD, but whether the same effect occurs in humans is unknown. Studies have observed the relationship between PD and COVID-19 and found that PD itself is not a risk factor for developing severe COVID-19,^[5,65]^ but cardiovascular disease and the elderly have been confirmed to be risk factors for new coronavirus infection.^[66,67]^ Therefore, there is a the pathological link between COVID-19 and PD remains controversial. Based on the mechanism by which COVID-19 affects the central nervous system and the pathogenesis of PD, this study explored the possible pathological link between the 2.

#### 1.3.1. *Will COVID-19 induce PD*?

To analyze whether COVID-19 induces PD, it is necessary to understand the impact of COVID-19 on the human body and its pathological mechanism, especially its impact on the central nervous system. Because the pathogenesis of PD is complex, it is necessary to consider the impact of COVID-19 on the human body as a whole and analyze how these effects are related to the brain and even PD, and whether it is consistent with the etiology and pathogenesis of PD.

Patients infected with SARS-CoV-2 often present with severe pneumonia. Similar to SARS coronavirus, the virus enters respiratory cells through the ACE2 receptor, and structural proteins play an important role in the budding process of viral particles released from different host cells^[68]^. White blood cells and lymphocytes decreased in patients with COVID-19; C-reactive protein and erythrocyte sedimentation rate were generally increased, creatine kinase, myoglobin, aspartate aminotransferase, alanine aminotransferase, lactate dehydrogenase, D-dimer, creatine phosphokinase, and platelet levels are elevated,^[69,70]^ and procalcitonin levels may be elevated during viral-bacterial co-infections. Studies have shown that macrophages and dendritic cells play a crucial role in the adaptive immune system, and these cells contain inflammatory cytokines and chemokines such as IL 6, IL 8, IL 12, TNF*α*, monocyte chemotactic protein 1, granulocyte-macrophage colony-stimulating factor, granulocyte-colony stimulating factor, and inflammatory factors that may contribute to systemic and neuroinflammation.^[68]^ It can be seen from the above that SARS-CoV-2 infection can cause neuronal cell death and neuroinflammation by changing the levels of cells, protein molecules and inflammatory factors, and the pathogenesis of PD is mainly related to neurodegeneration and neuroinflammation. SARS-CoV-2 may also infiltrate the CNS directly through the olfactory or vagus nerve, or through the hematopoietic pathway, which in turn promotes cytotoxic aggregation of proteins, including *α*-synuclein, in the striatum or accelerates neurodegeneration.^[71]^ Other studies have shown that neuronal populations are not equally susceptible to degeneration and that dopaminergic neurons are selectively vulnerable owing to their inherent properties. For example, the higher bioenergetic demands of highly dendritic axons and impaired protein homeostasis of larger axons can promote *α*-synuclein aggregation and lead to increased demand for *α*-synuclein proliferation. Selective susceptibility to discrete non-cell-autonomous factors such as neuroinflammation and environmental neurotoxins.^[71]^ Thus, SARS-CoV-2 infection may promote an increase in *α*-synuclein, which may exacerbate the original cell-autonomous vulnerability and lead to the spread of *α*-synuclein and extensive neurodegeneration. As discussed in the section on the impact of COVID-19 on the central nervous system, the homeostasis of the gut microbiota is disrupted after SARS-CoV-2 infection, promoting gut-produced *α*-synuclein through the brain-gut axis system. Aggregate in the brain can promote the production of neuroinflammation, etc, so COVID-19 may also indirectly affect brain function and become a risk factor for PD. Other studies have suggested that anti-PD drugs can treat COVID-19 to a certain extent,^[72,73]^ which may explain the pathological link between PD and COVID-19, and the 2 share some common pathological mechanisms. Dopamine may be involved in the pathophysiology of COVID-19 by regulating ACE2 and dopamine decarboxylase (DDC) synthesis.^[74]^ ACE2 receptor is highly expressed in dopamine neurons and is reduced in PD, which is characterized by dopamine deficiency.^[75]^ A study has shown that SARS-CoV-2-induced ACE2 expression defects may alter neurotransmitter levels in COVID-19 patients.^[6]^In addition, the toxic effects of SARS-CoV-2 infection trigger the stimulation of caspases-2, -3, and -8 through the NF-*κ*B pathway, leading to the death of dopamine neurons.^[76]^It has also been shown that after respiratory hypoxia in COVID-19 patients, reactive oxygen species are produced to cause oxidative stress response, leading to inflammation and immune response, thereby affecting cellular pathways,^[77]^ resulting in more dopamine neuron death, leading to the occurrence and development of PD. Therefore, SARS-CoV-2 infection can reduce the synthesis of dopamine synthetase (ACE2 and DDC) and promote the death of dopamine neurons to change the dopamine system, thus leading to PD.

It is not difficult to see that SARS-CoV-2 infection may induce PD to a large extent by promoting or aggravating neuroinflammation, neuronal death, *α*-synuclein aggregation, and disrupting intestinal flora homeostasis.

#### 1.3.2. *Is PD a risk factor for COVID-19*.

The innate immune response and the acquired immune response are activated in PD patients, and *α*-synuclein can aggregate and increase after immune stimulation, which may destroy the immune system,^[5]^ but this conclusion has not been confirmed so far, and future research needs to Better understanding of the interaction between alpha-synuclein and the immune system, but theoretically PD has the potential to be susceptible to SARS-CoV-2. In addition, PD can cause mitochondrial dysfunction and oxidative stress, which may also be risk factors for viral infection.^[78]^ Prevention of RNA virus progression is an immediate and major concern for individual health, but both elevated *α*-synuclein levels and long-term inflammation have been associated with Lewy bodies pathology and increased risk of PD.^[79]^ Some studies^[9]^ believe that the expression of *α*-synuclein in neurons of the peripheral nervous system can inhibit the invasion of the virus to the CNS and limit the replication of RNA viruses in the brain.^[10]^ Physiological *α*-synuclein also plays a role in immune cell recruitment and protection of the pro-inflammatory response to other infections.^[11]^ Therefore, the overexpression of *α*-synuclein in PD may prevent the invasion of neurons by SARS-CoV-2 and, to a certain extent, prevent the occurrence of COVID-19. This contradicts the conclusion that “theoretically PD has the possibility of being susceptible to SARS-CoV-2.” However, other studies have shown that while elevated *α*-synuclein expression may indeed have an effect against this RNA virus, the aggregated *α*-synuclein contained in Lewy bodies is unlikely to be effective in limiting RNA viruses. Copy.^[80]^ In PD, acetylcholine and dopaminergic pathways are blocked by degeneration of the substantia nigra and motor neurons.^[81]^ However, in the case of viral infections, acetylcholine levels have been shown to be affected throughout the development of the virus. In addition, cholinergic lymphocytes in direct contact with pulmonary macrophages showed elevated levels of acetylcholine, which may be caused by the SARS-CoV-2 virus,^[82]^ which also regulates lung inflammation,^[83]^ which is a characteristic clinical manifestation of COVID-19. In a study using a single directional inoculation of Taylor’s mouse encephalomyelitis virus into the substantia nigra of the mouse brain, viral RNA was observed to appear in the substantia nigra within 3 days after infection and further spread throughout the brain within 10 days, but no viral RNA was detected after 3 weeks.^[83]^ In this study, the stereo-localization coordinates were established by stereo-localization map and dye injection, which accurately tracked the process of RNA virus diffusion and disappearance. Therefore, it can be considered that these interactions between the substantia nigra and viral pathogens may support the PD Braak hypothesis, and the interaction between neurotransmitter pathways and viral infection provides a possibility for further studies on the relationship between COVID-19 and PD. Elderly age is a risk factor for COVID-19, so the effect of age on COVID-19 may mask the conclusion that PD is a risk factor for SARS-CoV-2 infection. PD patients present with motor symptoms and non-motor symptoms,^[84]^ and the impact on the human body is extensive and holistic. The body functions and the ability to resist diseases are weakened, and there are some common pathological mechanisms between PD and COVID-19.^[72,73]^Because ACE2 receptor is highly expressed in dopaminergic neurons, its expression is significantly reduced in PD. Therefore, some studies speculated that there may be a reasonable relationship between ACE2 expression changes and DDC dysfunction in COVID-19 infected patients, suggesting that PD patients may be more vulnerable to coronavirus attack and lung epithelial infection.^[85]^

Therefore, I believe that PD patients are susceptible to SARS-CoV-2 infection. However, the conclusion in this regard is currently controversial, and further research is needed to verify this.

### 1.4. The impact of COVID-19 on PD

Infection is a common cause of PD exacerbation, and although it is associated with brain dopamine metabolism, pharmacodynamic changes, and the direct effects of endotoxins,^[86]^ the mechanism is unclear. Deterioration of motor function may persist after systemic inflammation.^[87]^ Severe infections, such as COVID-19, may have a direct adverse effect on PD motor symptoms, but the effect on specific clinical features is currently unclear. The neurotropic properties of SARS-CoV-2 may underlie the exacerbation of chronic neurological diseases, such as PD, through pharmacodynamic changes (such as the interaction between dopamine and the renin-angiotensin system in the substantia nigra and striatum.) and a variety of mechanisms, including systemic inflammatory responses, aggravate PD.^[88]^ Studies have shown that PD symptoms worsen in both the early and late stages of infection in patients with COVID-19,^[65]^ and that patients with advanced PD have a higher mortality rate from COVID-19, which is associated with older age and longer disease duration.^[75]^ A community study^[88]^ looked at 141 PD patients infected with SARS-CoV-2 (excluding the possible deleterious effects of isolation and lockdown restrictions on some confounding factors affecting motor and non-motor symptoms in PD, such as reduced physical activity, increased stress, confusion, anxiety, and sleep disturbances), and found that COVID-19 can lead to significant deterioration of motor symptoms, motor-related disabilities, and activities of daily living; it also significantly exacerbated some non-motor symptoms, such as fatigue and attention deficit, and urinary problems such as urgency, incontinence, and increased nocturia, which can be explained by infections as well as increased motor fluctuations that are the result of pharmacokinetic problems, but cognitive impairment and autonomic function barriers have nothing to do with COVID-19.The observation time of this study was 3 months, the purpose of which was to reduce the influence of PD progression on clinical characteristics change and recall bias, and to ensure the accuracy of the trial. However, the follow-up was conducted by the neurologist through remote interview (video consultation or telephone), and the evaluation was not combined with objective examination such as clinical imaging, so the evaluation results were subjective to a certain extent and the reliability was reduced. Some studies have suggested that various non-motor symptoms of PD, such as respiratory dyskinesia, anxiety, aspiration pneumonia, dierrhea, hypoolfaction aggravated, and restrictive lung disease, may be related to covid-19 pathology.^[80]^COVID-19 and Parkinson’s disease pathology involve both innate and adaptive immune responses, so SARS-CoV-2 infection may aggravate the immunomodulatory response in PD patients and lead to more serious complications.^[85]^In conclusion, patients who died of COVID-19 during PD had a higher mortality rate than the general population, and both motor and nonmotor symptoms were aggravated by SARS-CoV-2 infection.

Owing to the impact of the epidemic, all countries have adopted epidemic prevention and control measures, such as isolation. PD is an irreversible neurodegenerative disease that relies on drug therapy and rehabilitation to relieve its symptoms and prevent disease progression. For PD patients, after isolation at home, the exercise mode, amount of exercise, and enthusiasm for exercise will inevitably be affected, or without the guidance of professional medical personnel, incorrect training methods and movements may occur, which will not only fail to improve motor symptoms but also aggravate them. Dysfunction or even unawareness of training precautions leads to problems such as falls, muscle damage, joint wear, and pain, which seriously hinder the recovery of motor function and the improvement of quality of life in patients with PD. In addition, the long-term isolation of the closed environment at home results in limited social participation in patients with PD, inability to find their correct life and social values, and worsening non-motor dysfunctions such as depression, anxiety, and insomnia that may already exist. This hinders the training of patients with PD and seriously affects their quality of life.

### 1.5. Telerehabilitation of PD patients

Due to the impact of the epidemic, PD patients have limited medical treatment and exercise training, so telerehabilitation is crucial. Telerehabilitation means that medical personnel use electronic devices and network platforms to conduct videos with patients and guide patients online to perform correct rehabilitation treatment.^[89]^ Telerehabilitation can formulate a rehabilitation treatment plan according to the current functional impairment of PD patients, supervise the patient’s training, fully mobilize the enthusiasm for treatment, and achieve the level of offline rehabilitation treatment as much as possible. Here is a brief overview of the principles, goals, methods and precautions of telerehabilitation for PD patients.

#### 1.5.1. *Principles of telerehabilitation*.

1. Safety is a priority, and if necessary, the patient’s family members are required to assist in protecting safety, and vital signs such as blood pressure, blood oxygen, and respiration should be detected in a timely manner.
2. effective communication, medical personnel conduct barrier-free and active communication with patients through online platforms so that medical personnel can keep an eye on the training status of patients at any time.
3. training feedback, patients should timely feedback their feelings after training to medical personnel, so that medical personnel can adjust the rehabilitation treatment plan in time.
4. functional assessment, medical personnel should respond to patients periodically to assess the functional level to understand the training effect and make a training plan suitable for the patient; and.
5. confidence is encouraged. Medical personnel should express their appreciation for the patients’ active training through online videos and praise the progress and improvement brought about by the training. The curative effect can improve the confidence and expectation of patients in rehabilitation, thereby improving their enthusiasm for training and treatment.
6. psychological counseling, medical staff, and patients can communicate online at any time, try to be friends with patients, understand the psychological and emotional status of patients, and timely elimination of negative emotions and actively guide patients to maintain an optimistic and healthy attitude.

#### 1.5.2. *The goals of telerehabilitation*.

The goal should be set according to the patient’s existing functional level, with specific reference to the hospital’s rehabilitation treatment goal setting, which can be divided into short- and long-term goals.

1. Short-term goals: On the basis of the patient’s current functional level, strengthen the current functions, and try to train the functions that are only available at a higher functional level, that is, strengthen the existing functions and induce nonfunctioning.
2. Long-term goals: Long-term goals can be used as a superposition of multiple short-term goals. The ultimate goal is to improve the quality of life and return to family and society, even if the patient’s motor function level is close to normal.

#### 1.5.3. *The methods of telerehabilitation*.

The method is the formulation of a rehabilitation treatment program, mainly referring to the motor function training program, which is formulated according to the Hoehn-Yahr stage. For specific rehabilitation methods, please refer to the European Parkinson’s Disease Physiotherapy Guidelines of the European Association of Parkinson’s Disease Physiotherapists (2014 edition).^[90]^ If these treatments cannot be performed under these conditions, alternative treatments can be sought, such as traditional Chinese exercises.

#### 1.5.4. *The precautions of telerehabilitation*.

The patients were trained in a relatively wide, uncluttered, flat, and well-lit environment; their family members were beside them to protect them. The heart rate was controlled within the range of (220-age) × (55%–65%), and the training intensity was based on heart rate detection. Adjustment: Loose clothing and slight sweating after training are appropriate to prevent wind.

## 2. Discussion

After the outbreak of COVID-19 in late 2019, many studies have found that COVID-19 can cause chronic diseases of the central nervous system.^[91]^ As the second most common neurodegenerative disease in the world, PD has received extensive research attention. The relationship between COVID-19 and PD has become a research hotspot. With further research, the relationship between the 2 has become increasingly controversial, including whether PD is a risk factor for SARS-CoV-2 infection and whether COVID-19 can cause PD. Based on the controversy surrounding this argument, this article summarizes the existing literature and further explains the possible relationship between COVID-19 and PD by analyzing the impact of COVID-19 on the central nervous system and the pathogenesis of PD. provides new information for their prevention and treatment. In the context of the pandemic, the principles, goals, methods, and precautions of remote rehabilitation of PD patients are briefly proposed, which compensates for the lack of home rehabilitation of PD patients, maintains the enthusiasm and continuity of patient training, and is conducive to functional rehabilitation and improvement of quality of life. Improve and reduce the burden of PD patients in hospitals in the context of an epidemic. The results of this study show that there is sufficient evidence that COVID-19 can induce PD, but whether PD is a risk factor for SARS-CoV-2 infection remains controversial, and more in-depth research is needed to verify this.

## Author contributions

**Writing – original draft:** Xiaoming Xi.

**Writing – review & editing:** Xiaoming Xi, Liang Han.
